# Dataset on potato phenology, growth, and yield for DSSAT/APSIM crop model calibration at central region of Bangladesh

**DOI:** 10.1016/j.dib.2025.112363

**Published:** 2025-12-05

**Authors:** A.H.M. Motiur Rahman Talukder, Faruque Ahmed, A.F.M. Shamim Ahsan, S.N. Mahfuza, A.A.M.M. Mustakim, Nadira Mokarroma, Lutfun Nahar, Md Shihab Uddine Khan, Zakaria Alam

**Affiliations:** a Plant Physiology Division, Bangladesh Agricultural Research Institute (BARI), Gazipur 1701, Bangladesh; b Horticulture Research Centre, BARI, Gazipu 1701, Bangladesh; c Agricultural Botany Division, Sher-e-Bangla Agricultural University (SAU), Dhaka 1207, Bangladesh; d Agricultural Research Station, BARI, Satkhira, 9400, Bangladesh; e Tuber Crops Research Centre, BARI, Gazipur 1701, Bangladesh

**Keywords:** Planting schedules, Total dry matter, Tuber growth rate, Potential tuber growth rate, Leaf area, Leaf growth rate

## Abstract

Understanding potato phenology under varying planting schedules is essential for optimizing planting practices, predicting harvest time, improving pest and disease management, and ensuring climate resilience. In Bangladesh, where potato is a priority crop in the winter season, determining the optimum planting schedules is critical to maximize yield potentiality. However, limited information exists on how shifts in planting schedules influence growth dynamics, phenology, and thermal indices of potato varieties under central region conditions. Respond to this problem, a field experiment was conducted during winter season in the central region of Bangladesh to evaluate the response of two potato varieties (BARI Alu-25 and BARI Alu-41) to four planting schedules at 10-day intervals (20 November, 30 November, 10 December, and 20 December 2023–2024). The trial was laid out in a Randomized Complete Block Design (RCBD) with three replications. The data compiled for this article such as crop Phenology (seedling emergence, stolon initiation, tuber initiation, and bulking), biological and economic yield, total dry matter (TDM), Leaf area index (LAI), leaf dry weight, leaf growth rate, leaf area expansion rate, tuber dry weight, potential tuber growth rate (PTGR) traits were collected as per schedule following the established methods and protocols. All collected data were analyzed using a computer-based software R. This dataset explored the impact of varying planting schedules on the local microclimate and how these changes affect potato development, growth patterns, and yield. It further determines the best pairing of potato varieties and planting schedules to achieve maximum production. This dataset captures potato phenology, growth, and yield under varying planting schedules in subtropical Bangladesh. These data will help researchers study potato growth and genotype × environment effects, and guide policymakers to set best planting schedules for sustainable production in South Asia.

Specifications Table

**Table:** 

Subject	Agricultural and biological science
Specific subject area	Agronomy and crop Science
Type of data	Raw, Analyzed, Table etc
Data collection	Key phenological stages (seedling emergence, stolon initiation, tuber initiation, and bulking of tuber) were recorded as days from sowing using three replicated plants, and mean values were analyzed. Physiological maturity was noted by leaf yellowing, after which potatoes were harvested. Green leaf area was measured using a LI-3100C meter to calculate the leaf area index and leaf area expansion rate. Leaves and tubers were dried at 80 °C for 72 h; dry weights used to compute leaf growth rate (LGR) and potential tuber growth rate (PTGR). Tuber yield (t ha^-1^) was determined from a 1.2 × 4.0 m² plot, including marketable and non-marketable tubers. Mean trait values were used for analysis.
Data source location	Plant Physiology Division, Bangladesh Agricultural Research Institute (BARI) in Gazipur, Bangladesh is the owner of the dataset presented in this manuscript. The experimental site is located (in between 23°53′ and 24°21′ N latitudes and 90°09′ and 92°39′ E longitudes), approximately 35 km north of Dhaka, the capital.
Data accessibility	Repository name: Mendeley Data DOI: 10.17632/4pt9tdjy3z.1 Direct URL to data: https://data.mendeley.com/datasets/4pt9tdjy3z/1
Related research article	None

## 1 Value of the Data

• This dataset provides a comprehensive field-based evaluation of potato phenology, growth, and yield responses to variable planting schedules under subtropical conditions of Bangladesh.
• The dataset is essential for calibrating and validating crop simulation models (DSSAT, APSIM), which are widely used to predict crop performance under climate change scenarios.
• Researchers can use these data to compare potato growth dynamics across agro-ecological zones and identify genotype × environment interactions.
• The data will be valuable for policymakers and agronomists in determining optimum planting schedule for sustainable potato production in South Asia.
• It provides a benchmark dataset for future meta-analyses and cross-location comparisons on potato thermal indices (GDD, HUE).

## 2 Background

Potato (*Solanum tuberosum* L.) is a key food and cash crop in Bangladesh, grown mainly during the rabi (dry) season with supplemental irrigation. The sequence of developmental stages from emergence to maturity together with overall crop growth is determined by genetic makeup and shaped by environmental influences such as temperature, photoperiod, and soil water availability. Process-based crop models like DSSAT and APSIM require detailed input data on phenology, biomass production, and yield to simulate potato growth and performance accurately. Optimizing phenological progress through suitable planting schedule is crucial for achieving higher yields and better tuber quality [1]. Under warmer early-season temperatures, earlier planting may extend the vegetative phase but can postpone tuber initiation [2]. Potato foliage grows best at 20–25 °C, while tuber bulking is favored by cooler conditions of 15–20 °C [[3], [4], [5], [6]].

Although potato phenology has been widely studied in temperate climates, there remains a knowledge gap regarding how varying planting schedule affect growth and yield under the climatic conditions of central Bangladesh. Naturally, the central part of Bangladesh experiences a typical tropical monsoon climate, marked by hot and humid summers and cooler dry winters. Average temperatures are around 25 °C, rising to 30–36 °C in April-May and dropping to about 10 °C in January. Most rainfall is about 80 % of the yearly total occurs during the monsoon from June to September. To address this, a structured dataset was compiled through field experiments in the region, documenting key developmental stages, growth dynamics, and yield components. These records provide reference data for model calibration and validation, facilitating the assessment of crop responses across various environmental circumstances. Ultimately, deriving valuable insights from model outputs breeders, producers and policymakers can formulate climate-resilient potato production strategies that maximize productivity and ensure national food security.

## 3 Data Description

This dataset was collected from a field experiment conducted during the winter season of 2023–2024 at the research field of the Plant Physiology Division, Bangladesh Agricultural Research Institute (BARI), Gazipur-1701. The site is in central Bangladesh (23°53′−24°21′ N, 90°09′−92°39′ E), about 35 km north of Dhaka, the capital. Table 1. explains the interaction effects of planting schedule and potato varieties on phenological (Days to emergence, stolon initiation, tuber initiation, tuber bulking, harvest, biological & tuber yield parameters in the central region of Bangladesh. In Table 2 presented total dry matter production of potato varieties under different planting schedules. In Table 3, Table 4, Table 5 & Table 6 presented Leaf area index (LAI), leaf dry weight, Leaf growth rate & leaf area expansion rate of potato varieties under different planting schedules. In Table 7 & Table 8 Tuber dry weight & Potential tuber growth rate of potato varieties under different planting schedules were presented.

**Table 1:** Phenology, days to harvest and yield of potato varieties under different planting schedules.

Planting schedules	Varieties	Days to emergence	Days to stolon initiation	Days to tuber initiation	Days to tuber bulking	Days to harvest	Biological yield (t ha^-1^)	Tuber yield (t ha^-1^)
20 November	BARI Alu-25	17.0 a	19.0 ab	27.0 b	31.0 b	92.0 b	27.48 c	26.39 bcd
	BARI Alu-41	14.0 b	17.0 de	30.0 a	32.0 a	102.0 a	39.47 a	37.07 a
30 November	BARI Alu-25	13.0 d	17.0 de	22.0 c	26.0 d	84.0 d	26.78 c	25.53 cd
	BARI Alu-41	12.0 e	16.0 e	27.0 b	31.0 ab	102.0 a	37.10 a	35.03 a
10 December	BARI Alu-25	14.0 bc	17.0 cd	19.0 d	22.0 e	80.0 e	25.35 cd	24.33 de
	BARI Alu-41	14.0 bc	18.0 bc	27.0 b	29.0 c	85.0 c	31.25 b	28.20 bc
20 December	BARI Alu-25	12.0 e	18.0 bc	22.0 c	25.0 d	79.0 f	22.43 d	21.36 e
	BARI Alu-41	13.0 cd	20.0 a	27.0 b	30.0 bc	85.0 c	31.43 b	29.46 b
CV ( %)		4.02	3.74	2.93	2.40	5.09	6.79	7.09
LSD_0.05_		0.95	1.17	1.29	1.20	0.722	3.58	3.53

**Table 2:** Total dry matter of potato varieties under different planting schedules.

Planting schedules	Varieties	Total dry matter (kg ha^-1^) of potato varieties at different days after planting (DAP)			
		30 DAS	45 DAS	60 DAS	75 DAS
20 November	BARI Alu-25	596.0 d	2229.4 bc	4213.4 e	7658.3 cd
	BARI Alu-41	852.0 c	3149.9 a	7070.9 ab	10,529.7 b
30 November	BARI Alu-25	962.5 b	3239.7 a	5150.5 d	7507.3 d
	BARI Alu-41	1081.8 a	2974.2 a	7386.2 a	13,642.6 a
10 December	BARI Alu-25	285.4 f	1915.7 cd	5128.0 d	8787.0 c
	BARI Alu-41	346.6 f	1769.5 d	5919.2 c	11,213.1 b
20 December	BARI Alu-25	297.3 f	1852.5 d	4193.3 e	8290.6 cd
	BARI Alu-41	457.1 e	2311.0 b	6523.4 bc	10,655.0 b
CV ( %)		6.63	7.44	6.11	7.01
LSD_0.05_		70.87	317.0	610.0	1201.3

**Table 3:** Leaf Area Index (LAI) of potato varieties under different planting schedules.

Planting schedules	Varieties	Leaf Area Index (LAI) at different days after planting (DAP)			
		30 DAP	45 DAP	60 DAP	75 DAP
20 November	BARI Alu-25	0.44 e	1.21 e	1.46 b	1.06 d
	BARI Alu-41	0.84 b	2.05 a	2.16 a	1.80 a
30 November	BARI Alu-25	0.70 c	1.30 d	1.35 bc	0.93 e
	BARI Alu-41	1.16 a	1.85 b	1.99 a	1.70 b
10 December	BARI Alu-25	0.24 f	0.79 f	1.13 d	0.92 e
	BARI Alu-41	0.43 e	0.84 f	2.00 a	1.49 c
20 December	BARI Alu-25	0.20 *g*	0.81 f	1.20 cd	0.64 f
	BARI Alu-41	0.51 d	1.52 c	2.05 a	1.86 a
CV ( %)		3.917	2.91	5.84	3.99
LSD_0.05_		0.0387	0.066	0.171	0.0909

**Table 4:** Leaf dry weight of potato varieties under different planting schedules.

Planting schedules	Varieties	Leaf dry weight (g m^-2^) at different days after planting (DAP)			
		30 DAP	45 DAP	60 DAP	75 DAP
20 November	BARI Alu-25	33.43 c	66.36 d	189.63 f	263.60 c
	BARI Alu-41	53.70 b	100.26 a	313.43 d	403.00 b
30 November	BARI Alu-25	54.23 b	72.06 cd	211.66 ef	272.36 c
	BARI Alu-41	64.73 a	82.53 b	413.76 b	483.23 a
10 December	BARI Alu-25	14.86 e	47.16 e	233.16 e	261.86 c
	BARI Alu-41	21.50 d	64.30 d	450.90 a	517.66 a
20 December	BARI Alu-25	13.46 e	52.96 e	181.26 f	196.86 d
	BARI Alu-41	25.96 d	81.83 bc	359.10 c	386.43 b
CV ( %)		8.80	8.33	6.16	9.17
LSD_0.05_		5.431	10.35	31.76	55.9

**Table 5:** Leaf growth rate of potato varieties under different planting schedules.

Planting schedules	Varieties	Leaf growth rate (g m^-2^ day^-1^) at different days after planting (DAP)		
		30–45 DAP	45–60 DAP	60–75 DAP
20 November	BARI Alu-25	2.19 c	8.21 e	4.93 a
	BARI Alu-41	3.11 ab	14.21 d	5.97 a
30 November	BARI Alu-25	1.19 d	9.31 e	4.04 ab
	BARI Alu-41	1.18 d	22.08 b	4.63 ab
10 December	BARI Alu-25	2.15 c	12.40 d	1.91 bc
	BARI Alu-41	2.85 bc	25.77 a	4.45 ab
20 December	BARI Alu-25	2.63 bc	8.55 e	1.04 c
	BARI Alu-41	3.72 a	18.48 c	1.82 bc
CV ( %)		17.33	9.14	9.90
LSD_0.05_		0.722	2.381	2.84

**Table 6:** Leaf area expansion rate of potato varieties under different planting schedules.

Planting schedules	Varieties	Leaf Area expansion rate (cm^2^ m^-2^ day^-1^)		
		45–30 DAP	60–45 DAP	75–60 DAP
20 November	BARI Alu-25	671.6 c	220.5 cde	−341.1 abc
	BARI Alu-41	1080.0 a	90.3 e	−320.4 abc
30 November	BARI Alu-25	538.5 de	46.2 e	−383.0 bc
	BARI Alu-41	614.8 cd	121.1 de	−250.5 ab
10 December	BARI Alu-25	495.5 e	301.4 bcd	−186.2 a
	BARI Alu-41	368.0 f	1035.7 a	−457.5 c
20 December	BARI Alu-25	549.8 de	347.1 bc	−493.1 c
	BARI Alu-41	892.9 b	464.2 b	−151.0 a
CV ( %)		7.83	9.24	−34.52
LSD_0.05_		89.36	185.40	195.2

**Table 7:** Tuber dry weight g m^-2^ of potato varieties under different planting schedules.

Planting schedules	Varieties	Tuber dry weight (g m^-2^) at different days after planting (DAP)		
		45 DAP	60 DAP	75 DAP
20 November	BARI Alu-25	110.2 e	399.2 bc	448.5 e
	BARI Alu-41	140.7 c	406.1 bc	505.8 de
30 November	BARI Alu-25	110.8 e	302.8 d	529.6 cd
	BARI Alu-41	162.3 a	412.7 b	676.7 a
10 December	BARI Alu-25	117.2 d	392.2 bc	583.1 c
	BARI Alu-41	71.0 h	489.5 a	552.4 cd
20 December	BARI Alu-25	103.8 f	386.9 c	527.7 cd
	BARI Alu-41	97.1 *g*	485.2 a	664.1 b
CV ( %)		2.73	3.04	6.655759
LSD_0.05_		6.06	21.8	66.8449

**Table 8:** Potential tuber growth rate of potato varieties under different planting schedules.

planting schedules	Varieties	Potential tuber growth rate (g m^-2^ day^-1^) at different days after planting (DAP)	
		60–45 DAP	75–60 DAP
20 November	BARI Alu-25	19.3 c	3.28 e
	BARI Alu-41	17.7 cd	6.64 de
30 November	BARI Alu-25	16.13 e	15.1 b
	BARI Alu-41	16.7 d	17.2 a
10 December	BARI Alu-25	18.3 c	12.7 bc
	BARI Alu-41	27.9 a	4.19 e
20 December	BARI Alu-25	18.8 c	9.38 cd
	BARI Alu-41	25.8 b	11.9 bc
CV ( %)		4.79	10.27
LSD_0.05_		1.58	4.036

## 4 Experimental Design, Materials and Methods

The field experiment was conducted during the winter season of 2023–2024 using two potato varieties, BARI Alu-25 and BARI Alu-41 obtained from Tuber Crop Research Centre (TCRC), BARI. The potato varieties were evaluated under four different planting schedules: 20 November, 30 November, 10 December, and 20 December 2023. The experiment was arranged in a Randomized Complete Block (RCB) factorial design with three replications, where the planting schedules were randomly assigned within each block. Each experimental unit measured 2.4 *m* × 3.0 m. Seed tubers of each variety were hand-planted at a spacing of 60 cm × 25 cm and a depth of 5 cm in furrows. Fertilizers were applied following the general recommendations of the Fertilizer Recommendation Guide-2018 (https://www.bfa-fertilizer.org/wp-content/uploads/2019/09/Fertilizer-Recommendation-Guide-2018-English.pdf) [7]. Earthing-up was performed simultaneously to protect tubers from sunlight exposure, promote bulking, and facilitate harvesting.

### 4.1 Crop data collected

#### 4.1.1 Phenological parameters

The time required for each major phenological stages *i.e.* seedling emergence, stolon initiation, tuber initiation, tuber bulking, and physiological maturity were recorded from the observation of three replications. Days to emergence was recorded when ∼90 % plant comes above the soil surface and maturity as the onset of leaf yellowing. Stolon initiation was tracked by sampling three plants plot^-1^ two to three times weekly from two weeks after emergence.

#### 4.1.2 Growth parameters

Green leaf area was measured using a leaf area meter (LI-3100C; Li-Cor, USA), and leaf area index (LAI) was computed accordingly. Leaf area per plant and biomass partitioning were measured at 30, 45, 60, 75 DAP, and at harvest.

$\text{Leaf}\;\text{area}\;\text{index}\;\left(\text{LAI}\right)=\;\frac{\text{Total}\;\text{green}\;\text{leaves}\;\text{area}\;({\text{cm}}^{2})}{\text{Area}\;\text{occupied}\;\text{by}\;\text{the}\;\text{plants}\;({\text{cm}}^{2})}$

$\text{Leaf}\;\text{expansion}\;\text{rate}\;\left(\text{LER}\right)=\;\frac{\Delta \text{LA}}{\Delta T}\;=\;\frac{\left(L_{2}-L_{1}\right)}{\left(T_{2}-T_{1}\right)}$ cm^2^ m^-2^ day^-1^

Where, LA represent the leaf area and T represent the time (day) respectively

Dry matter accumulation was determined from a 1.2 × 4.0 m² harvest area. Total plant biomass was separated into leaves, stems, roots, and tubers. The plant parts were dried in an oven for 72 h at 80 °C and dry weight was measured. From the weight of oven dried plant sample the leaf growth rate, potential tuber growth rate was measured by using following formula:

$\text{Leaf}\;\text{growth}\;\text{rate}\;\left(\text{LGR}\right)=\;\frac{\Delta W}{\Delta T}\;=\;\frac{\left(W_{2}-W_{1}\right)}{\left(T_{2}-T_{1}\right)}$ g m^-2^ day^-1^

Potential tuber g$\text{rowth}\;\text{rate}\;\left(\text{PTGR}\right)=\;\frac{\Delta W}{\Delta T}\;=\;\frac{\left(W_{2}-W_{1}\right)}{\left(T_{2}-T_{1}\right)}$ g m^-2^ day^-1^

Where, W and T represent weight (g) and time (day), respectively. tuber yield (t ha⁻¹) was determined from a 1.2 × 4.0 m² harvest area, including both marketable and non-marketable tubers.

Biologicalyield(t/ha)=Economicyield(tha ^-1^ )+Stoveryield(tha ^-1^ )

### 4.2 Statistical analysis

The collected data was subjected to analysis of variance (Two-way ANOVA) following the Randomized Complete Block Design. Mean separations were performed using the Least Significant Difference (LSD) test at a 5 % probability level whenever the F-test was significant. All statistical analyses were conducted using computer-based software “R” developed by [8]. Since the interaction effects were significant for all measured traits, the combined effects are reported.

## Limitations

The dataset on potato phenology, growth, and yield from the central region of Bangladesh provides valuable information for DSSAT/APSIM model calibration; however, some limitations was noticed. The dataset size is relatively small, as it was based on a single experimental trial, which may limit the robustness of model calibration and validation. Environmental variability such as unexpected rainfall, temperature fluctuations, or soil heterogeneity may have introduced uncontrolled variation in the recorded parameters. In addition, some growth and phenological measurements, particularly at intermediate growth stages, relied on periodic field observations, which may contain minor human error. Finally, the dataset focuses mainly on two potato varieties, limiting its applicability to a wider genetic pool.

## Ethics Statement

The dataset was generated through controlled field trials, with no involvement of human subjects or animals. All experimental activities were conducted following established ethical guidelines for plant research, prioritizing environmental sustainability and responsible resource use. The collected data are authentic, carefully documented, and made publicly accessible to support transparency, reproducibility, and future scientific investigations.

## CRediT authorship contribution statement

**A.H.M. Motiur Rahman Talukder:** Conceptualization, Methodology, Investigation, Supervision, Writing – original draft. **Faruque Ahmed:** Conceptualization, Methodology, Investigation, Supervision, Validation. **A.F.M. Shamim Ahsan:** Writing – review & editing. **S.N. Mahfuza:** Writing – review & editing. **A.A.M.M. Mustakim:** Data curation, Software. **Nadira Mokarroma:** Investigation, Supervision, Data curation. **Lutfun Nahar:** Data curation, Software, Writing – review & editing. **Md Shihab Uddine Khan:** Writing – original draft, Writing – review & editing. **Zakaria Alam:** Writing – review & editing.

## Data Availability

Mendeley DataPhenology, growth and yield of Bangladeshi potato varieties (Original data).
